# Allogeneic CAR Cell Therapy—More Than a Pipe Dream

**DOI:** 10.3389/fimmu.2020.618427

**Published:** 2021-01-08

**Authors:** Kenneth J. Caldwell, Stephen Gottschalk, Aimee C. Talleur

**Affiliations:** Department of Bone Marrow Transplantation and Cellular Therapy, St. Jude Children’s Research Hospital, Memphis, TN, United States

**Keywords:** allogeneic, CAR, cell therapy, immunotherapy, cancer

## Abstract

Adoptive cellular immunotherapy using immune cells expressing chimeric antigen receptors (CARs) has shown promise, particularly for the treatment of hematological malignancies. To date, the majority of clinically evaluated CAR cell products have been derived from autologous immune cells. While this strategy can be effective it also imposes several constraints regarding logistics. This includes i) availability of center to perform leukapheresis, ii) necessity for shipment to and from processing centers, and iii) time requirements for product manufacture and clinical release testing. In addition, previous cytotoxic therapies can negatively impact the effector function of autologous immune cells, which may then affect efficacy and/or durability of resultant CAR products. The use of allogeneic CAR cell products generated using cells from healthy donors has the potential to overcome many of these limitations, including through generation of “off the shelf” products. However, allogeneic CAR cell products come with their own challenges, including potential to induce graft-versus-host-disease, as well as risk of immune-mediated rejection by the host. Here we will review promises and challenges of allogeneic CAR immunotherapies, including those being investigated in preclinical models and/or early phase clinical studies.

## Introduction

Adoptive cellular therapy refers to the isolation of immune cells, followed by *ex vivo* manipulation and subsequent delivery into patients as a therapeutic intervention. An area of interest is the exploration of cellular or immunotherapeutic approaches for the treatment of oncologic diseases, including using chimeric antigen receptors (CARs) (1–3). CARs combine the specificity of an antibody with signaling domains of effector cells and costimulatory molecules (1–3). When constitutively expressed on the surface of an immune cell through non-viral or viral transduction, CARs enable an effector cell to recognize targets in an antigen-specific manner. CARs designed to target a specific tumor-associated-antigen (TAA) can then be used for anticancer therapy (1–3).

Cell therapy with T cells expressing CARs (CAR T cells) represent a significant advance in the field of cancer immunotherapy and is fueling the development of CAR-based immunotherapies using other immune cells. The most successful CAR cell therapy approach thus far has been the treatment of patients with highly relapsed/refractory CD19-positive hematological malignancies using CD19-CAR T cells derived from autologous T cells. Across numerous institutions, using a variety of CAR constructs and manufacturing strategies, CD19-CAR T cell therapy has been extremely efficacious (4, 5). This success led to the FDA approval of three such products: tisagenlecleucel (Kymriah, Novartis), axicabtagene ciloleucel (Yescarta, Kite Pharmaceuticals), and brexucabtagene autoleucel (Tecartus, Kite Pharmaceuticals) (6–9). Additionally, autologous CAR T cells have shown robust anti-tumor activity for hematological malignancies targeting BCMA, CD20, CD22, and CD30 (10–13).

The autologous (patient-derived) CAR T cell paradigm has also highlighted the limitations of such therapies, including the challenges of leukapheresis, manufacturing and efficacy in an often heavily pre-treated patient population (14). Seeking to overcome these barriers, allogeneic CAR strategies are actively being developed. Significant challenges of using allogeneic cells exist and center upon the inherent immunologic mismatch between donor and recipient. However, despite these challenges, allogeneic CAR strategies hold the potential to offer quicker, more efficacious and more accessible CAR therapies.

**Figure 1:**
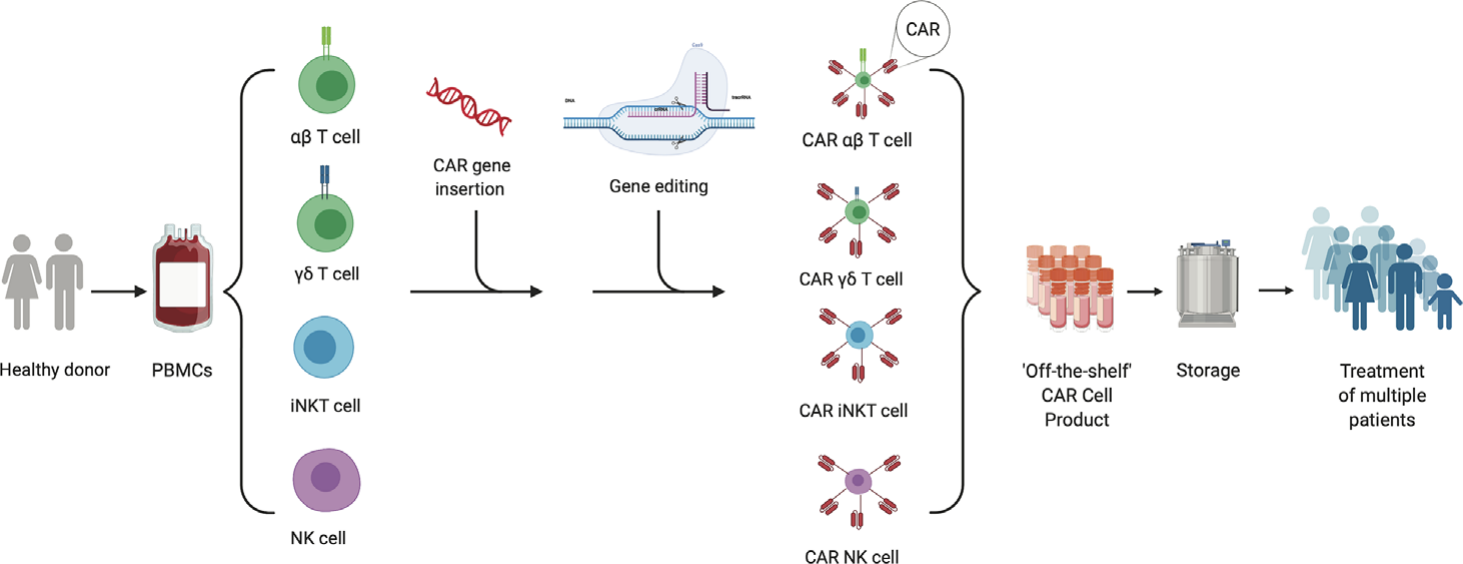
“Off the Shelf” allogeneic cellular therapy production. The production process for an allogeneic cellular therapy starts with collection of peripheral blood mononuclear cells (PBMCs) *via* leukapheresis from a health donor. Cells can then be sorted and selected for depending on the desired starting cellular material. CAR-encoding genes can be either inserted by non-viral or viral transduction or gene editing into immune cells. Additional gene editing can be performed to knock out genes of interest to mitigate risks such as immunogenicity and/or graft-versus-host-disease. The final product created from a single donor can be expanded, stored and used to treat multiple patients.

In this review, we will discuss a variety of allogeneic CAR cell therapy platforms that are being developed, including the use of different immune cells and/or subtypes, as well as gene-editing techniques (**Figure 1**). Additionally, we will highlight clinical experiences with allogeneic CAR cell therapies and on-going clinical trials to treat malignancies.

## The Need for Allogeneic CAR Therapies

Most CAR cell therapies to date, including the FDA approved products, are generated using autologous T cells. This has several important advantages, including infusion of CAR-engineered cell products without immunologic mismatch between donor and recipient. However, the use of autologous immune cells also has clinical and economical disadvantages. Autologous CAR cell production can be long and complicated. The process includes navigating the logistics of performing successful leukapheresis for a patient with relapsed/refractory malignant disease, accessing a manufacturing/treatment facility, and shipping and manufacturing times that commonly take several weeks. This time delay can be significant, particularly in a group of patients with aggressive relapsed/resistant cancers, who are at risk of clinical deterioration which could preclude proceeding with CAR cell therapy. Furthermore, generation of a cell product is not guaranteed and for those whom a product can be successfully generated, a proportion have limited short- or long-term efficacy. This is likely in part due to poor autologous immune cell fitness in cancer patients, particularly following aggressive cancer-directed therapies (15). Earlier collection of T cells may ameliorate some issues related to autologous T cell fitness. However, this strategy would only benefit the subset of patients determined to be at high-risk of needing CAR therapy early on in their disease process. Lastly, autologous cell therapy is performed for individual patients and is associated with significant costs, limiting broader applications of this therapy (16, 17).

**Figure 2:**
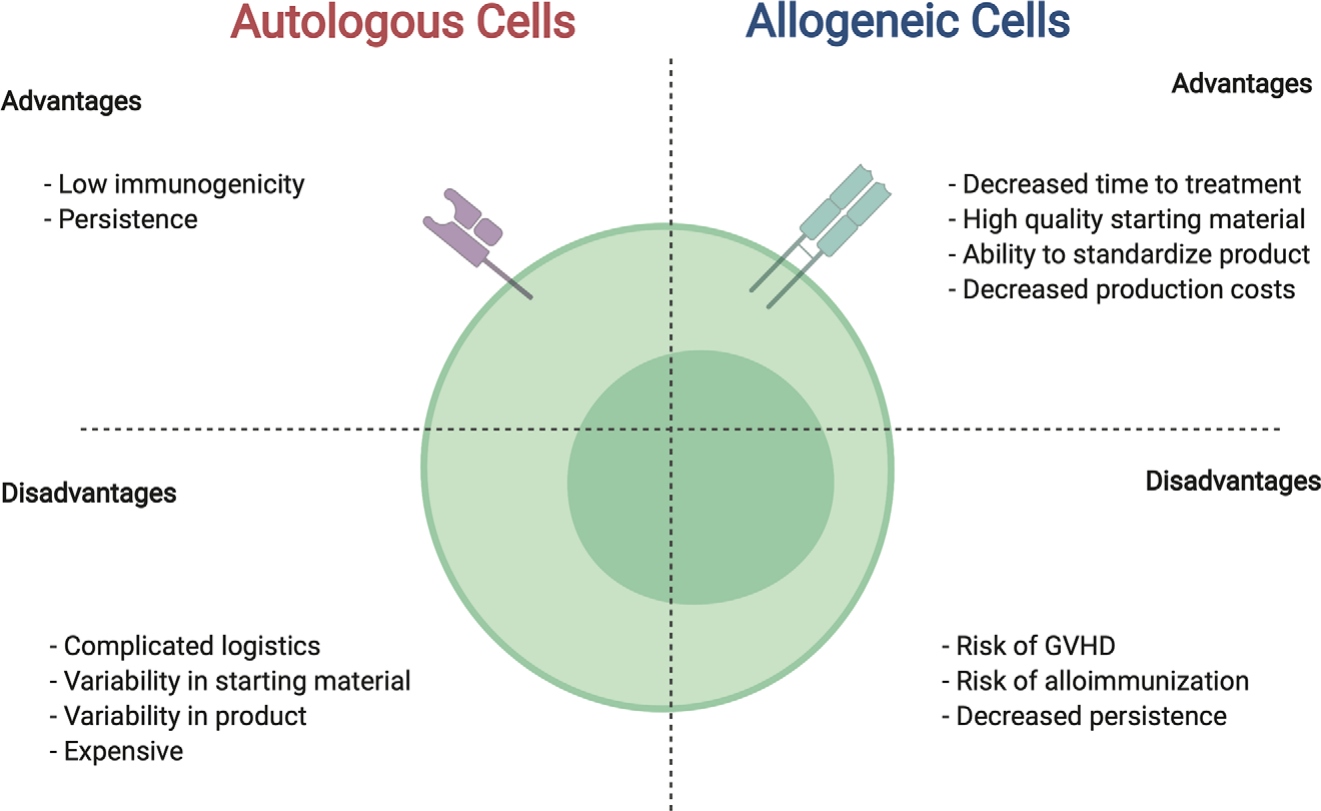
Comparison between autologous and allogeneic cells for use in CAR cell therapies. For details see text.

The use of immune cells from donors, or allogeneic cell therapies, offers many advantages over autologous cells including the potential to be cost effective, readily available, and provide a higher quality product (**Figure 2**). Healthy donor cells confer a more uniform starting material, allowing for more predictable manufacturing and performance of generated cell product. Following the single donor to single recipient model, use of a family member would provide an easily accessible and highly motivated allogeneic donor. Furthermore, allogeneic therapies have the potential to provide a ready to use “off the shelf” immunotherapeutic, such that a single manufacturing run would allow dosing for several patients and/or multiple dosing for individual patients. Likewise, by increasing the scale of production and creating an inventory or bank of manufactured CAR immune cells from healthy donors, the cost per patient would decrease while access to product would increase.

## Challenges with Allogeneic Therapies

Despite the recognized potential benefits of allogeneic CAR therapies, they are not without risk and significant challenges must be overcome in order to successfully implement this approach. These challenges stem from the immunologic mismatch between donor and recipient, and the resultant bidirectional risk to the cellular product and to the recipient *in vivo*. If the administered allogeneic cells recognize and attack healthy recipient tissues, the cellular therapy may cause unwanted graft-versus-host-disease (GVHD). Conversely, if the recipient’s immune system recognizes and reacts against the allogeneic product the cell therapy may be rejected, limiting the therapeutic effect.

### Graft-Versus-Host-Disease

Allogeneic hematopoietic cell transplant (HCT) highlights the significant risk of GVHD by adoptive transfer of allogeneic T cells (18). Human leukocyte antigen (HLA) mismatch between donor and recipient leads to donor immune recognition of, and subsequent alloreactivity against, recipient tissues (19–21). Clinically, this is manifested as GVHD. T cells are primarily responsible for causing acute GVHD, triggering tissue cell death *via* FAS ligand, perforin, granzyme and other signaling pathways (22, 23). The risk of GVHD correlates with increasing donor/recipient HLA-disparity. Most commonly affecting the skin, gastrointestinal tract and liver, GVHD carries a significant risk of post-HCT morbidity and mortality (24, 25). In HCT, the risk of GVHD may be mitigated through donor selection, T cell depletion/selection and/or use of immunosuppressive pharmacologic therapies (26–30). However, some of these strategies are in direct opposition with the goals of allogeneic CAR therapies which depend on highly immunocompetent cells. Therefore, decreasing the risk of GVHD from allogeneic CAR immune cells must balance with the need to retain high levels of immune activity of the effector cell. Such strategies may include T cell-subset selection or gene editing approaches, as well as continued exploration of cell products such as γδ T cells, invariant (i)NKT cells, or allogeneic NK cells that do not induce GVHD.

### Immunogenicity

Expansion and persistence of CAR immune cells are important to achieve both short- and long-term efficacy. While desired duration of persistence may vary based on the malignancy being treated, it has been shown that prolonged remission of acute leukemia correlates with duration of persistence of autologous CAR T cells (31). In the autologous setting, CAR cell longevity can be compromised through immunological rejection of the CARs “foreign” proteins. The use of allogeneic immune cells carries further increased risk of immunogenicity as both the CAR and effector cells are “foreign.” Acutely, this would result in impaired short-term responses as cells are rejected before exerting the intended therapeutic effect. The use of lymphodepleting chemotherapy prior to infusion of allogeneic CAR cellular products should mitigate the risk of acute rejection, augmenting CAR cell persistence (32). However, subsequent recipient immune reconstitution may result in delayed rejection of the adoptively transferred cells, providing an opportunity for malignant relapse. Furthermore, the use of allogeneic cells confers risk of alloimmunization, where the recipient develops donor-specific anti-HLA antibodies (DSAs). Alloimmunization is a well-recognized cause of graft failure and rejection in HCT (33). While desensitization strategies exist, the development of DSAs may preclude a patient from proceeding with HCT in the future or limit re-dosing of the allogeneic CAR product. Genetic modification to remove donor major histocompatibility complex (MHC) molecules or expansion of donor pools to allow for increased HLA-matching may mitigate these risks.

## Allogeneic CAR Strategies by Effector Cell Type

The most widely used CAR platform currently in clinical practice are CAR T cells. These products are largely manufactured using a batched pool of autologous donor T cells collected *via* peripheral blood leukapheresis and the CAR-T product administered without selection of specific cell types. While this strategy may work in the autologous setting, to mitigate potential risk of using allogeneic immune cells strategies using various T cell subgroups or different immune cell types are being tested in both preclinical and clinical settings. Here we review strategies being explored to make allogeneic CAR immune therapy possible, as well as on-going clinical trials evaluating these strategies (**Table 1**).

**Table 1:** Selected clinical studies with allogeneic CAR immune cells.

Target	Diagnosis	Strategy to reduce GVHD and/or rejection	Other genetic modification	NCT #
**T cells**				
BCMA	MM	TRAC KO	CAR (LV), CD52KO	NCT04093596
	MM	TRAC and B2M KO	CAR (knock in)	NCT04244656
CD7	T-cell leuk or lymph	TRAC KO	CAR (*), CD7 KO	NCT04264078
CD19	NHL	TRAC KO	CAR (LV), CD52KO	NCT03939026
	NHL	TRAC KO	CAR (LV), CD52KO	NCT04416984
	B-ALL	TRAC KO	CAR (LV), CD52KO	NCT02808442
	ALL	*	CAR (*)	NCT04173988
	B-cell leuk or lymph	TRAC and B2M KO	CAR (LV)	NCT03166878
	B-cell leuk or lymph	TRAC and B2M KO	CAR (knock in)	NCT04035434
	B-cell leuk or lymph	*	CAR (*)	NCT04384393
	B-cell leuk or lymph	*	CAR (*)	NCT04264039
	Leuk or lymph	*	CAR (*)	NCT04227015
	B-ALL or lymph	TRAC KO	CAR (knock in)	NCT03666000
	B-cell leuk or lymph	*	CAR (*)	NCT03229876
	B-ALL	*	CAR (*)	NCT04166838
CD19/20/22	Leuk or lymph	TRAC KO	CAR (*)	NCT03398967
CD20	Lymph	*	CAR (*)	NCT04176913
	B-cell lymph or CLL	*	CAR (knock in)	NCT04030195
CD22	B-ALL	TRAC KO	CAR (LV), CD52KO	NCT04150497
CD70	Leuk or lymph	TRAC and B2M KO	CAR (knock in)	NCT04502446
	RCC	TRAC and B2M KO	CAR (knock in)	NCT04438083
CD123	AML	TRAC KO	CAR (LV)	NCT03190278
CS1	MM	TRAC KO	CAR (LV)	NCT04142619**
NKG2DL	CRC	*	CAR (RV)	NCT03692429
Mesothelin	Mesothelin+ ST	TRAC KO	CAR, PD1 KO	NCT03545815
**EBV-specific T cells**				
CD19	Leuk or lymph	Cell product	CAR (RV)	NCT01430390
CD30	Lymph	Cell product	CAR (RV)	NCT04288726**
**γδ T cells**				
NKG2DL	ST	Cell product	CAR (RV)	NCT04107142**
**iNKT cells**				
CD19	B-cell leuk or lymph	Cell product	CAR (RV), IL15	NCT03774654
**NK cells**				
CD19	B-cell leuk or lymph	Cell product (cord blood)	CAR (RV), IL15	NCT03056339
CD19	B-cell lymph or CLL	Cell product (iPSC)	CAR, IL15, CD16	NCT04245722

ALL, acute lymphoblastic leukemia; CLL, chronic lymphatic leukemia; CRC, colorectal cancer; NHL, non-Hodgkin lymphoma; MM, multiple myeloma; Leuk, Leukemia; Lymph, lymphoma; RCC, renal cell carcinoma; ST, solid tumor.

LV, lentivirus; RV, retrovirus; *not disclosed; **not yet recruiting or currently closed for recruitment (as of 10’2020).

### T Cells

T cells are a powerful component of the human immune system, providing surveillance for, and protection against, foreign antigen. Antigen recognition occurs *via* the T cell receptor (TCR), a heterodimer complex composed of two subunits and located on the surface of T cells. In a healthy donor, the majority (> 90%) of circulating T cells have a TCR consisting of an alpha (α) and beta (β) chain, referred to as an αβ T cell (34). The remaining T cells contain a TCR composed of a gamma (γ) and delta (δ) subunit, γδ T cells (35). The interaction between the TCR and antigen triggers a signaling cascade through the TCR which activates the T cell. αβ T cells recognize foreign or non-self-antigen presented through the MHC on antigen presenting cells, while γδ T cells are MHC-independent (36). Furthermore, αβ T cells can be subdivided based on function (i.e., CD4-positive and CD8-positive T cells) and/or degree of differentiation (i.e., naïve and memory T cells) (37, 38). When a T cell is transduced with a CAR, the CAR adds an additional receptor to the T cell without interruption to the native TCR. CAR T cells can expand and contract in response to antigen stimuli *via* the CAR, allowing for robust responses in the setting of active target recognition, but also potential for memory-surveillance state when an intended target is not currently present. Below, we review different types of T cells used to generate allogeneic CAR T cells.

#### T Cells From Prior Allogeneic Transplant Donor

The use of donor lymphocyte infusion (DLI) after allogeneic HCT is a standard clinical practice. The therapeutic intent of unmodified DLI centers on the properties of donor T cells, such that they can correct mixed donor/recipient chimerism and combat viral infections (39). However, DLIs are not specific for TAAs and therefore have minimal anti-cancer benefit, especially outside the setting of minimal residual disease (39–41). As CAR T cells began to be explored clinically for the treatment of active disease, an initial venture into allogeneic CAR T cell products focused on the post-HCT population using T cells from the HCT donor. Brudno et al. evaluated the use of allogeneic CD19-directed CAR T cells derived from an individual patient’s HCT donor to treat patients with progressive disease after transplant, who had a median donor chimerism of 100%, demonstrating anti-tumor benefits and safety of this approach, including no reports of new-onset GVHD (42). This study exemplified the possibility of increasing the potential for graft-versus-tumor effect of donor-derived T cells, without significantly increasing risk of GVHD.

The use of HCT-donor-derived CAR T cells is limited to post-HCT patients with an available and willing donor, whom are largely treated at facilities with the capability to manufacture clinical grade CAR T cell products. Therefore, this method is innately lacking some of the benefits of an “off the shelf product” and is not widely accessible. However, several benefits of using an allogeneic product are retained, including the use of healthy donor cells, ease of leukapheresis timing and minimal risk of diminished persistence *in vivo* due to lack of HLA-mismatch. Additionally, this approach allows for exploration into the use of CAR T cells in different disease settings such as prophylaxis post-HCT to reduce relapse in high-risk populations. Data suggest that this strategy is feasible without added toxicity and, in addition to providing leukemic control, may help with control of viral-reactivations post-HCT *via* native TCR recognition (43, 44).

#### Virus-Specific T Cells

The adoptive transfer of allogeneic virus-specific T cells (VSTs) has emerged as a safe and effective means of providing antiviral benefit in multiple patient populations (45–48). This has led to the generation of partially HLA-matched banks comprised of libraries of “off the shelf,” purified allogeneic VSTs. Importantly, across numerous clinical studies including in allogeneic HCT populations, the incidence of GVHD has been very minimal. Although the complete mechanism is not fully understood, decreased TCR diversity in VSTs (i.e., memory T cells) is felt to decrease the risk of alloreactivity (i.e., GVHD).

The safety profile seen using allogeneic VSTs created interest in the development of an allogeneic platform using CAR-transduced VSTs. Demonstrating feasibility of VSTs as effector cells, autologous CAR transduced VSTs targeting TAAs (CD30 [Hodgkin lymphoma], HER2 [glioblastoma] and GD2 [neuroblastoma, osteosarcoma]) have successfully been manufactured and infused into patients, with encouraging safety and efficacy profiles (49–51). This strategy has also been explored in the post-HCT patient population using primarily donor-derived VSTs and thus far results are promising. In one study, CD19-redirected VSTs were generated using peripheral blood mononuclear cells (PBMCs) collected from the HCT donor and then infused into patients with B-cell malignancy at escalating doses (52). Manufacturing time for this product was significant, requiring culture for 5 – 6 weeks. Treatment was well tolerated with no GVHD and there was evidence of anti-leukemia activity, as well as retained recognition of viral stimuli. Similar preliminary results have been reported in an on-going trial evaluating allogeneic EBV-specific T cells transduced with a CD19-CAR (NCT 01430390) (53). Notably, donor sources in this trial include the HCT donor or 3^rd^ party donors when the HCT donor is not available, with recipients of the CAR-transduced 3^rd^ party cells also showing encouraging response rates.

Clinical experience to date with allogeneic CAR-transduced VSTs has shown intended anti-tumor effects with minimal GVHD risk. Additional benefit includes the finding that viral-specificity is retained and can trigger CAR T cell expansion *in vivo*, thereby potentially providing on-going, intermittent stimulus and promoting persistence. A limitation of studies thus far centers on the fact that data are largely confined to CAR-transduced VSTs derived from a patients’ HCT donor, thereby drastically minimizing the challenges of rejection and alloimmunization. Drawing from clinical experiences with unmodified VSTs from 3^rd^ party banks, persistence of VSTs is typically limited to a few months (48); therefore, we would hypothesize that the issue of rejection and limited persistence of CAR-transduced VSTs remains.

#### Memory T Cells

When devising strategies for allogeneic cellular therapies the use of memory T cell subsets as effector cells may confer a decreased risk of GVHD. T cell maturation and differentiation inversely correlates with alloreactivity, such that memory T cell subsets are less alloreactive than naïve T cells. Therefore, memory T cells are less likely to cause GVHD in the HLA-mismatched setting (54). Functionally it has also been noted that the effectiveness and persistence of CAR T cells is influenced by the degree of differentiation of the T cell subsets in autologous CAR T cell platforms (55–58). While the use of memory T cells to generate autologous CAR T cells is actively being studied using a variety of CAR constructs (NCT03389230, NCT02146924, NCT02051257, NCT03288493), their use in the allogeneic setting has not yet been evaluated.

T cell subsets can be distinguished through identification of extracellular surface markers, including CD45RO, CD45RA, CD62L, CCR7 and CD27 (37, 38). Several studies have highlighted that generating CAR T cells from central memory (CD45RO+/CD62L+ or CCR7+) T cells or memory stem cells (Tscm) T cell populations is associated with improved CAR T-cell effector function (59–61). Other groups have just focused on utilizing CD45RA-negative T cells, which includes the central memory and effector memory T cell subsets, since these subsets have decreased alloreactive potential (62). After showing promise in animal models, CD45RA-depletion began to be studied in human allogeneic HCT. Clinical studies demonstrated that this approach is feasible and carried a decreased risk of GVHD, both when utilized in primary graft manipulation and post-transplant DLI (63–67). Building upon this clinical experience, the role of memory T cell subsets as effector cells in CAR therapy has been studied pre-clinically. Investigators have shown that CD45RA-negative T cells expressing either a NKG2DL-specific or CD19-CAR have anti-cancer effects and decreased *in vivo* and *in vitro* alloreactivity (68–70). Using a similar approach, CD19-CAR-engineered CD27-negative T cells (effector and terminal effector memory subsets) have also shown promise in preclinical models (71). These data suggest that the approach of using allogeneic memory T cells as effector cells in CAR therapy may have merit in the clinical arena; however, additional studies are needed to define the optimal memory T cell subset, which should be used as a source to generate memory CAR T cells with reduced alloreactivity.

#### Genetically Modified αβ T Cells

**Figure 3:**
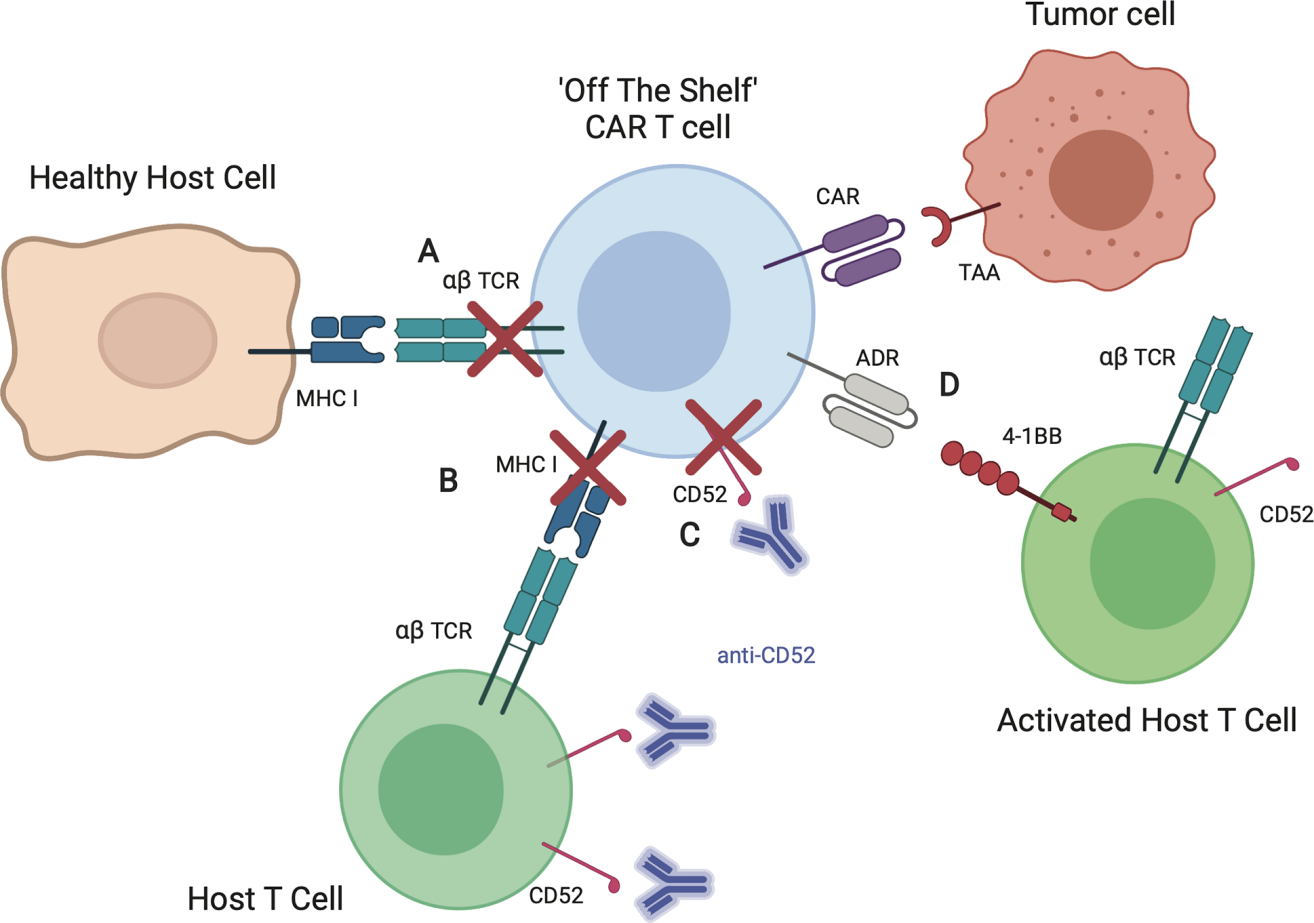
Strategies to improve allogeneic CAR T cells. **(A)** Elimination of the αβ TCR by knockout of TRAC or retaining TCRs within the Golgi apparatus reduces the risk of allogeneic T cell recognition of healthy host tissues, thereby decreasing risk of graft-versus-host-disease (GVHD). **(B)** Elimination of MHC I on allogeneic T cells by knockout of β-2 microglobulin (B2M) reduces the risk of host T cell recognition and elimination CAR T cells, increasing likelihood of allogeneic CAR T cell persistence. **(C)** Knockout of CD52, a T cell marker, allows for the use of CD52 antibody (anti-CD52) for enhanced lymphodepletion of host T cells without affecting infused allogeneic CAR T cells. **(D)** Expression of alloimmune defense receptor (ADR) destroys alloreactive host T cells targeting 4-1BB, decreasing the risk of rejection.

Several strategies are being explored to improve allogeneic αβ T cells, which are summarized in **Figure 3**. Gene editing of T cells to reduce the risk of GVHD and rejection is perhaps the most promising and widespread approach, particularly for the development of an “off the shelf” product. Given that GVHD is driven in large part by TCR recognition of host tissue, gene-editing approaches focused on the native αβ TCR of the effector cell are under investigation. Many groups have explored disrupting the T cell receptor constant alpha chain (TRAC) or beta chain (TRBC). Torikai et al. showed that knocking out the αβ TCR from CD19-CAR T cells did not significantly alter the cells ability to kill CD19-positive targets (72). This initial report in 2012 used zinc finger nuclease mediated knockout of TRAC or TRBC. In recent years, with advancement in gene editing techniques, numerous groups have demonstrated knockout of TRAC using transcription activator like effector nucleases (TALENs) as well as CRISPR/Cas9 (73, 74). Another technique, targeting the CAR to the TRAC locus, was associated with improved anti-tumor activity in one preclinical study (74). In addition to gene-editing approaches, protein-based strategies are being developed to retain the TCR within the Golgi apparatus using an anti-TCR linked to the KDEL motive (75). While these techniques have become increasingly efficient, any remaining T cells that continue to express αβ TCR can be removed magnetically *ex vivo* using anti- αβTCR antibodies. *Stenger et al.* showed that TCR knockout of CD19-CAR T cells had high anti-leukemic functionality in the absence of alloreactivity. However, the gene-edited CAR T cells did not persist as long *in vivo* compared to CAR T cells with endogenous TCR, demonstrating a possible concern with this technique (76). Furthermore, modification to the endogenous TCR does not address the issue of immunogenicity.

To decrease immunogenicity, investigators have targeted β-2 microglobulin (B2M), a component of HLA class I molecules that is present on all T cells (77). As recipient T cell recognition of allogeneic CAR T cells can occur *via* interaction of HLA/MHC, knockout of the B2M gene in CAR T cells may prevent alloantigen presentation by infused the T cells. This thereby offers another strategy to limit host recognition and clearance of infused allogeneic CAR T cells. Ren et al. showed that CAR T cells including knockout of B2M had reduced alloreactivity *in vivo* (78). Kagoya et al.** showed that B2M knockout to eliminate HLA class I as well as knockout of class II major histocompatibility complex transactivator (CIITA) to eliminate HLA class II improved CAR T cells persistence *in vitro* (79). Other strategies to mitigate immunogenicity rely on depleting resident immune cells. Lymphodepleting chemotherapy prior to cellular infusion by itself should reduce immune-mediated clearance of CAR T cells, however immune responses to components of the CAR have been reported (80–82). To increase immunosuppression post-CAR T cell infusion, investigators have explored the use of the monoclonal antibody, alemtuzumab, which targets the pan-lymphocyte antigen CD52 (83). Since the mean half-life of alemtuzumab is 6.1 days (84), this approach would require the knockout of CD52 on allogeneic CAR T cells to prevent their depletion post infusion (85). Since the use of alemtuzumab is associated with an increased risk of viral reactivation/infection (e.g. cytomegalovirus, adenovirus) after allogeneic HCT (86, 87), close monitoring of recipients of alemtuzumab and CAR T cells is warranted for now since the risk of viral reactivation/infection in the setting of CAR T cell therapy is currently unknown. Lastly, active depletion of alloreactive host T cells is being explored including the expression of so called alloimmune defense receptors (ADRs) on infused allogeneic T cells that selectively recognizes 4-1BB, a cell surface receptor that is temporarily upregulated by activated host T cells (88).

Several clinical trials using gene-edited allogeneic CAR T cells are actively enrolling with some early results presented in abstract format. Many of these products include knockout of both CD52 and TRAC, to address both risk of GVHD and rejection. Pooled data from the CALM (NCT 02746952) and PALL (NCT02808442) studies (cellular product: UCART19) were presented by Allogene Therapeutics and Servier. UCART19 features an anti-CD19 scFv, as well as TRAC and CD52 knockout and was first shown to have clinical activity in two infants with B-ALL who achieved molecular remission after treatment (89). Data presented included 17 patients with relapsed/refractory B-ALL treated with a lymphodepleting regimen including alemtuzamab, 14 of whom had a CR or CRi. Additionally, UCART19 showed overall an acceptable safety profile (90). Neelapu et al.** presented early results from the ALLO-501 trial (NCT03939026), which evaluates an allogeneic CAR T cell product targeting CD19 with knock out of TRAC and CD52, and includes a novel CD52 antibody for lymphodepletion prior to CAR T cell infusion. Twenty-two patients had been enrolled, with response seen in 12 of 19 evaluable patients (7 complete responses) (91). Wang et al.** presented the first 5 patients treated with TruUCART GC027, an allogeneic anti-CD7 CAR T cell product with knock out of TRAC and CD7 by CRISP/Cas9 gene editing technology to avoid both GVHD and fratricide. Four of the initial 5 treated patients showed a complete response with an acceptable safety profile (92).

Gene editing has emerged as the leading strategy being tested in the clinic as investigators seek to develop an “off the shelf” allogeneic CAR therapy platform. As gene editing techniques advance, this method offers great potential to mitigate potential risks and downsides associated with the use of allogeneic cells. By increasing the number of genetic edits made to a single cell the possibilities increase; however, it will take time to thoroughly investigate the short- and long-term safety of these gene edited products in patients.

#### γδ T Cells

Animal models show that γδ T cells play an important role in tissue homeostasis and cancer immunosurveillance (93). Allogeneic γδ T cells have been given to patients with cancer after lymphodepleting chemotherapy and were shown to expand *in vivo* without causing GVHD (94). γδ T cells recognize cancer through a broad spectrum of receptors, rather than in a single clonally expanded fashion, which may mitigate tumor escape *via* single antigen loss (95). These T cells are also typically abundant in tissues, which may provide an advantage over αβ T cells when developing therapeutics to treat solid tumors (93). Their ability to recognize targets in an MHC independent manner confers a low risk of alloreactivity and GVHD, thereby increasing the potential of using allogeneic γδ T cells in CAR T cell therapies.

Polyclonal γδT cells transduced with a CD19-CAR have been shown to expand and demonstrated anti-tumor effects *in vitro* and in *in vivo* murine models (96). **Capsomidis et al. demonstrated CAR dependent antigen specific killing *in vitro* using GD2-CAR γδ T cells (97). Several companies are now moving forward with clinical trial development using allogeneic CAR-transduced γδ T cells including, Adicet Bio, Cytomed Therapeutics, GammaDelta Therapeutics and TC BioPharm (98). Furthermore, a clinical trial evaluating allogeneic CAR γδ T cells targeting NKG2DL for the treatment of solid tumors has been registered on clinicaltrials.gov, but is not yet recruiting (NCT04107142).

### iNKT Cells

Invariant natural killer T (iNKT) cells are a rare subclass of immune cells that are restricted by CD1d, a glycolipid presenting HLA I like molecule expressed on B cells, antigen presenting cells and some epithelial tissues (99–101). Thus, since iNKT express an invariant TCR, they do not cause GVHD. iNKT cells have been shown to be decreased in number and defective in cancer patients (102–104). iNKT cells also protect from GVHD after allogeneic HCT (105–107). Preclinical studies of iNKT engineered with CARs targeting CD19 and GD2 have been effective in murine models against lymphoma and neuroblastoma, respectively (108–110). CAR-engineered iNKT cells also appear to be safe in humans and are promising for “off the shelf” use given the lack of GVHD. Preliminary results from an ongoing trial (NCT03294954) using autologous GD2-CAR iNKT cells with co-expression of IL15 for the treatment of pediatric patients with neuroblastoma have shown this approach to be feasible and safe (111). Clinical experience of allogeneic CAR iNKT cells is yet to be published so possible adverse events cannot be predicted, however an ongoing clinical trial evaluating allogeneic CAR19-iNKT cells for the treatment of hematological malignancy (NCT03774654) aims to test safety and efficacy.

### NK Cells

NK cells, are of great interest in the treatment of cancer as they contribute to graft-versus-tumor effect and do not cause GVHD (112, 113). Endogenous NK cells are part of the innate immune system and can target cancer cells that downregulate HLA class I molecules (112). Tumor cells often downregulate their HLA molecules as an escape mechanism against T cells, making them susceptible to NK cells (114–116). Clinically, adoptively transferred non-CAR engineered allogeneic NK cells have shown to be safe in patients with cancer (117–120). NK cells are a promising alternative to T cells for CAR engineering given the low risk of GVHD and their innate anti-cancer properties. Numerous preclinical studies have shown CAR engineered NK cells to be effective against hematologic malignancy targets (CD19 and CD20), as well as solid tumor targets (WT1 and GD2) (121–126). Notably, Liu et al.** published results on 11 patients with lymphoid tumors, treated on an early phase clinical trial using allogeneic (cord blood derived) NK cells transduced with a gene containing a CD19-CAR, IL-15, and an inducible caspase 9 safety switch. In this study, 73% of patients demonstrated anti-tumor response. Inclusion of IL-15 in the CAR construct may have contributed to the persistence of the NK cells, which were shown to expand and persist for at least 12 months. There were no major toxic effects of the therapy (127).

Allogeneic NK cells can be prepared form numerous sources, including peripheral blood mononuclear cells (PBMCs), cord blood, and pluripotent stem cells (iPSCs) (128). In addition, a NK-cell line, NK-92, genetically modified to express a CAR, is actively being explored in early phase clinical testing (NCT03383978) (129). Current strategies to generate clinical grade NK cells from PBMCs or cord blood rely on the use of irradiated feeder cells, most commonly K562 cells genetically modified to express i) 4-1BBL and membrane-bound (mb) IL15 or ii) mbIL21 and exogenous IL2 (130–132). More recently, exosomes or plasma membrane particles derived from mbIL21 expressing K562 cells have also been successfully used for the *ex vivo* expansion of NK cells (133). iPSCs present an attractive source for generating NK cells without feeder cells (134, 135), and an early phase clinical study with unmodified iPSC-derived NK cells is in progress (NCT03841110). In addition, iPSC cells can be genetically modified and/or gene edited prior to NK cell differentiation, enabling the provision of an unlimited supply of modified NK cells (136–138).

## Discussion

Autologous CAR T cell therapy has revolutionized the treatment of hematological malignancies, highlighting the therapeutic potential of cellular therapies, as well as opportunities for continued improvement. Subsequently, the number of CAR therapy trials has increased dramatically in recent years, exploring new targets, manufacturing strategies, CAR constructs, patient populations and effector cells. While many of these trials continue to use autologous immune cells, the number using allogeneic CAR products are rapidly increasing. The apparent benefits of allogeneic therapies have spurred a robust interest in developing techniques that counter the predicted limitations, including exploration of various effector cell types and gene editing techniques. One hurdle that will definitively need to be addressed is immunogenicity, which already has emerged as a potential roadblock of autologous CAR T cell therapies, especially when no lymphodepleting chemotherapy is given prior to T cell infusion (139). Thus, recipients of “off the shelf” cell products might require immune-modulation post cell infusion to enable their long-term persistence. However, given the extensive experience with allogeneic HCT and solid organ transplantation, we believe that immunogenicity will not present an unsurmountable barrier. While production of “off the shelf” therapeutic products require increased resources during the development and manufacturing process, the ultimate goal is to develop cell products that have a favorable safety and efficacy profile and are widely accessible and affordable. However, at present it is too early to estimate the cost of an allogeneic cell product; this will depend on the required genetic modifications, which might include not only viral transduction but also gene-editing. Another driving factor of cost will be how many cell doses can be prepared from one lot of “off the shelf” cell products since release testing of genetically-modified cell products is cost intense. Nevertheless, we believe that continued investment in the optimization of these allogeneic strategies is warranted based on the current data and that allogeneic cell products will usher in a new era of cell therapy.

## Author’s Note

Figures created with BioRender.com.

## Author Contributions

KC, SG, and AT wrote, reviewed, and edited the manuscript. All authors contributed to the article and approved the submitted version.

## Funding

The work of the authors is in part supported by the National Cancer Institute/National Institutes of Health grant P30CA021765. The content is solely the responsibility of the authors and does not necessarily represent the official views of the NIH.

## Conflict of Interest

SG has patent applications in the field of immunotherapy, is a DSMB member of Immatics, and on the scientific advisory board of Tidal.

The remaining authors declare that the research was conducted in the absence of any commercial or financial relationships that could be construed as a potential conflict of interest.
